# Phage-mediated horizontal transfer of a *Staphylococcus aureus* virulence-associated genomic island

**DOI:** 10.1038/srep09784

**Published:** 2015-04-20

**Authors:** Bo Youn Moon, Joo Youn Park, Sun Yung Hwang, D. Ashley Robinson, Jonathan C. Thomas, J. Ross Fitzgerald, Yong Ho Park, Keun Seok Seo

**Affiliations:** 1 Department of Basic Sciences, College of Veterinary Medicine, Mississippi State University, Mississippi state, MS 39762, United States; 2 Department of Microbiology, College of Veterinary Medicine and BK21 Program for Veterinary Science, Seoul National University, Seoul 151–742, South Korea; 3 Department of Microbiology, University of Mississippi Medical Center, Jackson, MS 39216, United States; 4 The Roslin Institute and Edinburgh Infectious Diseases, Royal (Dick) School of Veterinary Studies, University of Edinburgh, Midlothian EH259RG, United Kingdom

## Abstract

*Staphylococcus aureus* is a major pathogen of humans and animals. The capacity of *S. aureus* to adapt to different host species and tissue types is strongly influenced by the acquisition of mobile genetic elements encoding determinants involved in niche adaptation. The genomic islands νSaα and νSaβ are found in almost all *S. aureus* strains and are characterized by extensive variation in virulence gene content. However the basis for the diversity and the mechanism underlying mobilization of the genomic islands between strains are unexplained. Here, we demonstrated that the genomic island, νSaβ, encoding an array of virulence factors including staphylococcal superantigens, proteases, and leukotoxins, in addition to bacteriocins, was transferrable *in vitro* to human and animal strains of multiple *S. aureus* clones via a resident prophage. The transfer of the νSaβ appears to have been accomplished by multiple conversions of transducing phage particles carrying overlapping segments of the νSaβ. Our findings solve a long-standing mystery regarding the diversification and spread of the genomic island νSaβ, highlighting the central role of bacteriophages in the pathogenic evolution of *S. aureus*.

S*taphylococcus aureus* is a versatile pathogen and causes a wide range of diseases in humans and animals by producing an array of factors involved in virulence and niche adaptation^1^. Genome sequencing analysis showed that *S. aureus* genomes are highly variable as only ~75% of the gene content is shared by all strains^2^. The genomic plasticity of *S. aureus* is primarily attributed to mobile genetic elements (MGEs) such as prophages, plasmids, pathogenicity islands of *S. aureus* (SaPIs), and genomic islands (νSa), which have an array of genes encoding proteins involved in antibiotic resistance, virulence, and other contingency functions^2^,^3^,^4^. Some MGEs are widely distributed among most *S. aureus* strains, while others are strongly associated with certain clonal complexes, presumably due to barriers such as DNA restriction-modification systems and niche separation decreasing opportunities for horizontal transfer^2^,^3^,^5^.

The genomic island referred to as νSaβ (also known as SaPI3/m3) is located upstream of a tRNA gene cluster, and contains genes encoding a bacteriocin, hyaluronate lysase, serine proteases, bi-component leukotoxin D and E, and the enterotoxin gene cluster (egc)^6^. Extensive variation in virulence gene content has been observed at the νSaβ locus in different strains (Supplementary Figure S1)^2^,^7^,^8^. Moreover, recent population genetic work has identified hot spots for homologous recombination in the *S. aureus* chromosome centered on insertion sites of mobile elements, including ICE*6013*, SCC*mec* and νSaα^9^. However, the mechanisms underlying the mobilization of genomic islands νSaα and νSaβ are unknown.

## Results

### Sequence analysis of νSaβ in the strain RF122

**Figure 1:**
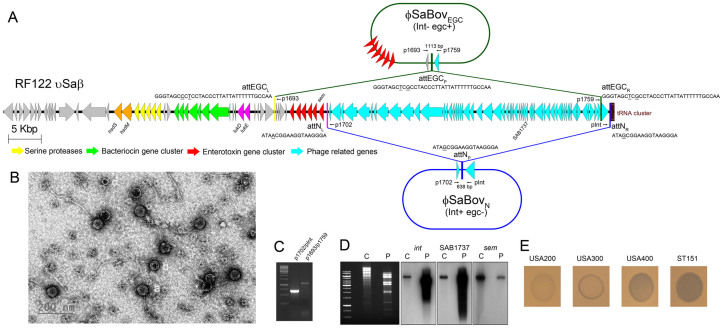
Heterogeneous excision products of the phage (φSaBov) that integrates at genomic island νSaβ. (A) A schematic map of νSaβ in the strain RF122. The arrows represent genes annotated in the GenBank entries^10^ and colored based on key features. Orange; restriction modification system HsdR/M, yellow; serine protease cluster (*spl*), light green; bacteriocin gene cluster (*bsa*), pink; leukocidins (*lukD/E*), red; enterotoxin gene cluster (*egc*), cyan; genes related to phage. Direct repeat sequences associated with phage and those associated with the egc were annotated as attN_L_ and attN_R_ and attEGC_L_ and attEGC_R_, respectively. Sequence variations in the direct repeats were underlined. Primers used for outward PCR and sequencing results of attN_P_ and attEGC_p_ were depicted. (B) Transmission electron microscope analysis of phage particles induced from the strain RF122. At least, three different head sizes (a, b, and c; 58, 47, 26 nm, respectively) of phages were observed. (C) Results of outward PCR using pInt/p1702 and p1693/p1759 for φSaBov_N_ and φSaBov_EGC_, respectively. (D) RF122 chromosomal DNA (C) and phage DNA (P) were digested with *EcoR*I, separated by electrophoresis, and transferred to Nylon membrane for Southern blot analysis. Probes specific to the integrase gene (SAB1760, for φSaBov_N_), SAB1737 (for φSaBov_N_ and φSaBov_EGC_), and the *sem* gene (for φSaBov_EGC_) were used. (E) Phage spot test. Mitomycin C induced culture lysate from the strain RF122 (10^8^ pfu/ml) was dropped onto the lawn culture of human ST36-SCCmecII (USA200), ST8-SCCmecIV (USA300), ST1-SCCmecIV (USA400), and bovine mastitis isolate (ST151).

The strain RF122 is a bovine mastitis strain which belongs to the CC151 lineage^10^. Genome sequence analysis of the RF122 revealed that a prophage (designated as φSaBov in this study), belonging to serogroup B, integrase group Sa8, and holin group 438^11^, is integrated adjacent to νSaβ between an upstream tRNA cluster and downstream of the egc locus, flanked by 18 bp imperfect direct repeats, designated as attN_R_ and attN_L_, respectively, with a single SNP (Fig. 1A). The attN_R_ is highly conserved in all sequenced *S. aureus* strains as it is a part of the tRNA-Ser gene^10^,^12^. Additionally, 33 bp imperfect direct repeats were found upstream of the *int* gene (attEGC_R_) and upstream of the *seg* gene (attEGC_L_). The attEGC_L_ is conserved in all sequenced strains harboring the egc in the νSaβ^10^,^13^. Of note, attEGC_R_ is highly conserved upstream of the *int* gene in 43 staphylococcal phage sequences available from NCBI GenBank and recognized by sigma factor H, a transcriptional regulator of phage related genes^12^.

### Phage induction and analysis of phage DNA

The presence of two different sets of direct repeats suggests that transducing phage particles induced from the strain RF122 may harbor heterogeneous phage DNA. To test this possibility, phage was induced by mitomycin C treatment. Electron microscopy demonstrated that induced phage has a long non-contractile tail typical of the *Siphoviridae* family^14^ with various sizes of hexagonal heads (Fig. 1B). To identify circularized forms of phage DNA, outward PCR and sequencing were performed. The pIntF/p1702R primer set generated an approx. 652 bp amplicon. Sequencing of this fragment revealed that phage DNA was circularized between attN_R_ and attN_L_, resulting in attN_P_, which was identical to attN_R_, presumably using the Campbell mechanism^15^ (Fig. 1A and C). This type of transducing phage particle only harbors typical genes related to phage, and referred to as φSaBov_N _(Int+, egc-). The other primer set p1759/p1693 generated an approx.1115 bp amplicon. Sequencing of this fragment showed that another phage DNA was circularized between attEGC_R_ and attEGC_L_, resulting in attEGC_P _which was identical to attEGC_R_ (Fig. 1A and C). This type of transducing phage particles harbors the egc and typical genes related to phage except the *int* gene, and referred to as φSaBov_EGC _(Int-, egc+). As controls, outward PCR using chromosomal DNA as a template did not show any amplicons (data not shown). Southern blot analysis showed that probes specific to the integrase gene (a marker for φSaBov_N_), SAB1737 (a marker for φSaBov_N_ and φSaBov_EGC_), and the *sem* (a marker for φSaBov_EGC_) gene were specifically bound to their corresponding targets (Fig. 1D). The RF122 strain also harbors a truncated phage DNA represented by genes from SAB0258 (integrase gene) to SAB0266. We investigated the excision of this segment in the phage DNA using PCR but no mobilization was detected, indicating this phage is inactive (data not shown). To ensure *S. aureus* chromosomal DNA was not contaminating in the phage DNA preparation, an excessive amount of exogenous chromosomal DNA was added to phage induced lysates, followed by RNase and DNaseI treatment, prior to phage DNA extraction and tested by PCR (Supplementary Figure. S2). It is noteworthy that the band intensity of the *sem* gene was weaker than those of the integrase or SAB1737 gene, suggesting φSaBov_N_ is more dominant than φSaBov_EGC_. The relative copy number of φSaBov_EGC_ compared to that of φSaBov_N_ was determined by calculating the relative copy number of the *sem* gene (specific to φSaBov_EGC_) to the *int* gene (specific to φSaBov_N_) in the phage DNA using quantitative real-time PCR and found to be 0.06215 ± 0.001, indicating approx. 6 of 100 phages are φSaBov_EGC_. A phage spot test was performed to evaluate the host range of transducing phage induced from the strain RF122; The test resulted in a clear zone of lysis in human isolates ST1-SCCmecIV (USA400) and a bovine mastitis isolate (CC151) and, to a lesser degree, in ST36-SCCmecII (USA200), and ST8-SCCmecIV (USA300) (Fig. 1E).

### Phage-mediated horizontal transfer of νSaβ

**Figure 2:**
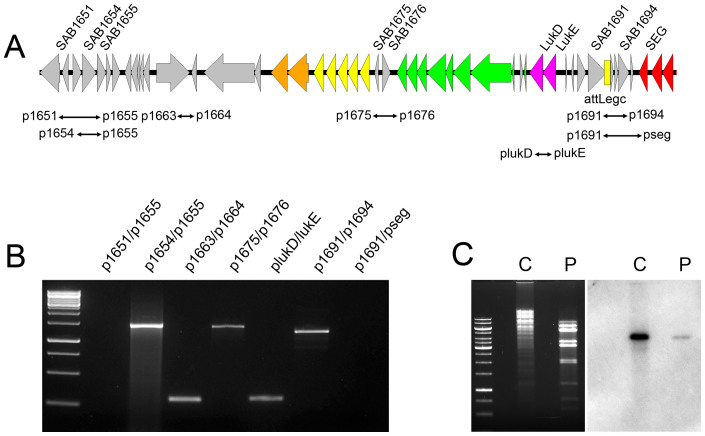
Identification of a tranducing phage particle, φSaBov_LUK_, harboring linear phage DNA. (A) A schematic map of linear phage DNA, based on PCR results (see below). Coloring of genes is as in Fig. 1. (B) Based on genome sequencing results of MNKN and CTH96 transductants, various sets of primer (see above map) were designed and tested to locate a linear form of phage DNA containing a bacteriocin gene cluster and LukD/E genes. PCR was positive with primer pairs p1654/p1655 and p1691/p1694 but not with p1651/p1655 and p1691/pseg, indicating a linear form of phage DNA with left flanking near SAB1654, and right flanking near SAB1694. (C) Southern blot analysis of RF122 chromosomal DNA (C) and phage DNA (P) digested with *EcoR*I restriction enzyme using a probe specific to the *lukE* gene (the membrane used in this figure is the same as in Fig. 1).

Mitomycin C treatment of strain RF122 can induce heterogeneous transducing phages harboring the egc, and these induced phages have a broad host specificity range, suggesting the egc could be transferred to other *S. aureus* by this phage. To test this possibility, the *tetM* gene, conferring tetracycline resistance, was introduced into the *sem* gene of the egc, resulting in RF122 *sem*::*tetM*. The phage induced from this strain was successfully transduced to various recipients. Similar to phage spot results, the transduction frequency to bovine (ST151) and USA400 (ST1-SCC*mec*IV) strains was much higher than those to USA300 and USA200 strains (Table 1). To further confirm the transfer of the egc, a draft genome sequence of the recipients MNKN (ST1-SCC*mec*IV) and CTH96 (CC151), and phage transduced strains (transductant) was determined. Strikingly, it was shown that both transductants have an identical sequence with the donor strain RF122 from the 141 bp downstream of the start codon of the SAB1676 gene (*bsaG*) to the attN_R_ sequence at the tRNA-Ser, even preserving SNPs at direct repeats, totaling to 65,756 bp. This result indicates that not only the integrase gene (from φSaBov_N_) and the egc (from φSaBov_EGC_), but also the region upstream of the egc containing a bacteriocin gene cluster and leukotoxin D/E genes, were transferred (Supplementary Figure S3). Southern blot analysis using a probe specific to the *lukE* gene demonstrated the presence of the transducing phage particle harboring the region upstream of the egc containing a bacteriocin gene cluster and leukotoxin D/E genes (Fig. 2C). To test whether this type of transducing phage particle also carries a circular form of phage DNA, outward PCR using various sets of primers was attempted from freshly prepared phage DNA templates and repeated more than 10 times but failed (data not shown). We then investigated the possibility of the existence of a linear form of phage DNA. Indeed, PCR was positive with primer pairs p1654/p1655 and p1691/p1694 but not with p1651/p1655 and p1691/pseg (Fig. 2B), suggesting a linear form of phage DNA with left flanking near SAB1654 and right flanking near SAB1694 (Fig. 2A). However, one cannot rule out the possibility that several intermediates might be detectable as a result of imperfect excision of φSaBov_N_ or φSaBov_EGC_ or a stochastic event (e.g. nucleases digested at the ends of the linear DNA that was possibly fragmented by the phage). This type of transducing phage particle harboring a bacteriocin gene cluster and leukotoxin D/E genes was designated as φSaBov_LUK. _To confirm the transduction activity of φSaBov_LUK_**,** the *tetM* gene was introduced at the *lukE* gene (RF122 *lukE*::*tetM*). The phage induced from this strain was also successfully transduced the *lukE* gene to various recipients with a much lower transduction frequency (Table 1).

### The role of integrase and terminase in the transfer of the νSaβ

The phage DNA excision, package, and integration are controlled by cooperative actions of integrase, excisionase, terminase, and host-encoded DNA binding proteins^15^,^16^,^17^. To examine the role of these genes from φSaBov on the transfer of the νSaβ, the *cat* gene, conferring chloramphenicol resistance, was inserted into the integrase (SAB1760) and, separately, into the terminase large subunit (TerL, SAB1726) gene in the RF122 *sem*::*tetM* strain. The mitomycin C treatment of these strains still induced a clear lysis within 3 hours, indicating that disruptions of these genes did not affect phage induction. However, outward PCR and PCR analysis showed that a disruption of the *terL* gene completely abolished the phage DNA packaging (Supplementary Figure S4) and complementation of the *terL* gene restored phage DNA packaging (data not shown), suggesting phage DNAs were packaged through headful packaging mechanism by terminase^18^. In contrast, disruption of the integrase gene did not affect phage DNA excision and circularization (Supplementary Figure S4). However, none of the transducing phage particles induced from this strain was transduced to the recipient strains and the complementation of the integrase gene restored transducibility (data not shown). These results suggest that the integrase encoded in the φSaBov is not required for the phage DNA excision and packaging but is required for phage DNA integration into the recipient chromosome. Furthermore, mitomycin C treatment of the RN4220 strain just carrying φSaBov_N_ induced excision and circularization of φSaBov_N_ phage DNA but a similar treatment of the MW2 strain did not (Supplemental Figure S5), indicating the excision and circularization of the φSaBov_N_ phage DNA is dependent on host background. Strain RF122 harbors 5 alternative integrase genes associated with other MGE such as SaPIm4, SaPI122, SaPIBov1, or two inactivated phages. Currently, we are investigating the restoration of the phage DNA excision and circularization in the MW2 strain carrying the φSaBov_N_ by complementation with an alternative integrase gene in the RF122.

### Postulation of the νSaβ transduction model

**Figure 3:**
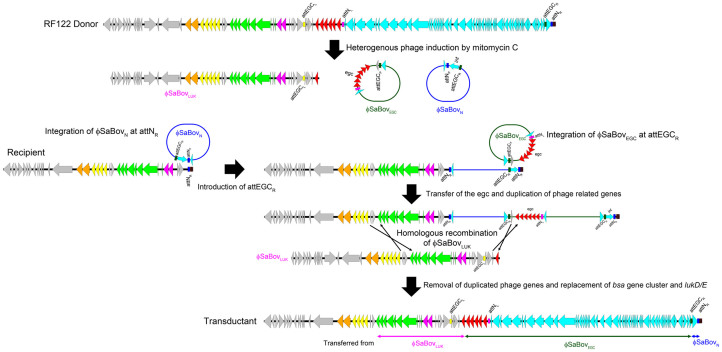
Proposed model for transfer of νSaβ mediated by φSaBov. Upon induction by mitomycin C, phage DNA (φSaBov_N_, φSaBov_EGC_, and φSaBov_LUK_) were excised from the RF122 chromosomal DNA and packed into phage head by terminase encoded in φSaBov. Upon entry to recipient strains, φSaBov_N_ phage DNA is firstly integrated into recipient host chromosomal DNA through recombination between attN_P_ (from φSaBov_N_) and attN_R_ (recipient chromosomal DNA). This event introduces the attEGC_R_ in recipient chromosomal DNA which allows φSaBov_EGC_ phage DNA for integrating into recipient chromosomal DNA through recombination between attEGC_P_ (from φSaBov_EGC_) and attEGC_R_ (recipient chromosomal DNA). This event generates duplication of phage DNA. Homologous recombination occurs between φSaBov_LUK_ phage DNA and integrated phage DNA, resulting removal of duplicated phage DNA. As a result of triple conversions, nearly all of the νSaβ from the donor strain is transferred to the recipient strain.

Considering these data, we postulate the following νSaβ transduction model (Fig. 3). φSaBov_N_ is firstly integrated into the attN_R_ sequence at the tRNA-Ser which introduces the attEGC_R_ site upstream of the int gene. Then, φSaBov_EGC_ is integrated into the attEGC_R_, resulting in the transfer of the egc and the duplication of the region spanning between attN_L_ and attEGC_R_. Homologous recombination events occur upstream of the SAB1676 gene, and downstream of attEGC_R_ with the linear phage DNA introduced by φSaBov_LUK_, resulting in the removal of the duplicating region spanning between attN_L_ and attEGC_R_ and the replacement of the region spanning the *lukE* gene, similar to Panton-Valentine leukocidin-phage mediated homologous recombination events between direct repeats of the two paralogous genes adjacent to the phage integration site^19^. As a result, nearly all of the νSaβ (from the 141 bp upstream of the start codon of SAB1676 gene to the attN_R_ sequence at the tRNA-Ser, a size of 65,767 bp) from strain RF122 was transferred to the recipient. Supporting this model, we were able to isolate transductant strains carrying intermediated forms of transduction carrying the φSaBov_N_ at tRNA cluster and the φSaBov_EGC_ at attEGC_R_ without homologous recombination of the φSaBov_LUK _using a junction PCR as shown in Supplemental Figure S6. Furthermore, transductant strains carrying the φSaBov_N _or both φSaBov_N_ and φSaBov_EGC_ exhibited an increased capacity to accept the φSaBov_EGC_ or the φSaBov_LUK_, respectively, as shown in supplemental Table S3.

### Distribution of a prophage in the νSaβ

To understand the significance of prophage in the dissemination of νSaβ, the prevalence of νSaβ and prophage in a collection of bovine isolates was investigated. From a collection of 2010–2013 bovine skin and mammary gland isolates from 8 different farms in the Ohio state, USA (n = 53), the presence of νSaβ was common (52/53, 98.1%) and 9 isolates (17.0%) have phage insertion between the egc and tRNA cluster, similar to the RF122 strain (Supplemental Figure S7). *spa* and MLST typing of these isolates showed that 7 and 2 isolates belong to CC97 and CC151, respectively, (Supplementary Table S4) which are commonly observed clonal complexes among ruminants^20^. By contrast, from another collection of bovine mammary gland isolates from 16 different farms in the Washington state, USA from 1985 to 2001 (n = 207), νSaβ was rare (102/207, 49.3%) and none of the isolates has the phage insertion at the νSaβ (data not shown). These results suggest that phage localization adjacent to νSaβ may have an important role in wide dissemination of the νSaβ in certain clonal complexes of bovine isolates.

## Discussion

The versatile host adaptation and successful pathogenicity of *Staphylococcus aureus* is strongly influenced by the acquisition of virulence factors encoded in the mobile genetic elements such as prophages, plasmids, pathogenicity islands, and genomic islands. The genomic island, νSaβ, is found in almost all *S. aureus* strains and is characterized by extensive variation in virulence gene content^2^,^7^,^8^. However the basis for the diversity and the mechanism underlying mobilization of the genomic islands between strains are unexplained. This is the first experimental evidence demonstrating the transfer of the genomic island, νSaβ, by the naturally occurring staphylococcal temperate phage, φSaBov. Remarkable features of φSaBov are that it generated heterogeneous transducing phage particles harboring circular and linear forms of phage DNA containing overlapping segments of the νSaβ, totaling to 65.7 kb, and sequentially integrated into the host chromosome by specific recombination events. The exact mechanism of linear phage DNA excision and site specific homologous recombination still remain elusive. Given the high transduction frequency of φSaBov to the epidemic human and animal isolates and the rapid spread of the νSaβ in the isolates from bovine mastitis with concurrent existence of phage insertion at the νSaβ, our findings highlight the importance of bacteriophages in the pathogenic evolution of *S. aureus* and the need for caution in the therapeutic use of phage as it may cause undesirable consequences, such as transfer of potent toxins and other virulence factors.

## Methods

### Bacterial strains and growth conditions

Strains used in this study were summarized in supplementary table S1. A collection of 207 *S. aureus* bovine mammary gland isolates (16 different farms, Washington state, USA) from 1985 to 2001, and 53 bovine mammary gland and skin isolates (8 different farms, Ohio state, USA) from 2010 to 2013 were kind gifts from Drs. Fox (Washington State University) and Rajala-Schlultz (Ohio State University), respectively. Multilocus sequence typing and *spa* sequence typing was done for nine isolates from a collection of Ohio state isolates harboring phage insertion in the νSaβ, using previously described methods^21^. *S. aureus* strains were typically grown in tryptic soy broth (TSB) or agar (TSA), with the supplementation of tetracycline (5 μg/mL) or chloramphenicol (10 μg/mL) when necessary.

### Phage induction and transduction

Cultures were grown to mid-log phase at 37°C with shaking (200 rpm), then mitomycin C (1 µg/mL) was added. The mixtures were incubated at 30°C with 80 rpm until complete lysis occurred (approximately 3 hours). The lysates were sterilized with syringe filers (0.22 µm). A phage spot test and the plaque forming unit (pfu) was determined by soft agar (0.5%) overlay method.

For transduction experiments, the recipient strains were cultured to mid-log phase and adjusted to approximately 2 × 10^7^ CFU/mL. A phage solution containing approximately 10^8^ PFU/mL was added to the culture, and incubated for 30 min at 30°C for the phage absorption, followed by adding sodium citrate solution (100 mM, pH 4.5). After centrifuging at 4,000 rpm, 4°C for 15 min, the pellet was resuspended in sodium citrate solution and plated on TSA supplemented with appropriate antibiotics.

### Transmission electron microscope (TEM) analysis of phages

Phage particles were placed on carbon-coated copper grids and washed briefly on water droplets. After washing, grids were dried and mounted with 2% uranyl acetate for 1 min and analyzed using TEM (Philips CM200).

### Phage DNA extraction and PCR

The mitomycin C treated culture lysates were treated with excessive amounts of RNase and DNase I (Sigma-Aldrich, 100 unit each), and then phage particles were precipitated with NaCl (0.5 M final concentration) and polyethylene glycol 8000 (10%, wt/vol), followed by ultracentrifugation at 100,000 × g for 1 h. Phage DNA was extracted using DNeasy kit (Qiagen) according to the manufacturers' instructions.

### PCR and quantitative real time PCR

All primer pairs used in PCR and outward PCR were listed in supplementary table S2. Quantitative real time PCR was performed to estimate relative copies of φSaBov_EGC_ to φSaBov_N_ using SYBR Green master mix (Applied biosystem) by calculating ΔC_T_ of the *sem* gene to the integrase gene in the phage DNA, according to the manufactures' instructions.

### Southern blot hybridization

Chromosomal and phage DNA were digested with *EcoR*I and resolved by electrophoresis in 0.5% agarose gels and transferred onto nylon membranes. Digoxigenin (DIG)-labeled DNA probes were synthesized using PCR-DIG DNA labeling kit (Roche) according to the manufacturers' instructions and primers listed in supplementary table S2. DNA hybridization and probe detection was performed using Chemiluminescent detection kits (Roche) according to the manufacturers' instructions.

### Allelic exchange constructs

All primer pairs used in allelic exchange constructs were listed in supplementary table S2. Allelic exchange, resulting in the insertion of antibiotic markers and target gene inactivation, was done using temperature sensitive pMAD system^22^ with minor modifications. The tetracycline resistance gene (*tetM*) and chloramphenicol resistance gene (*cat*) were amplified from the strain Mu50^23^ and pMK4 and cloned into the pMAD, resulting in pMAD-tet and pMAD-cat, respectively. The upstream and downstream gene fragments of target genes were amplified, and cloned into pMAD-tet or pMAD-cat. Resulting plasmids were electroporated into the strain RF122. Results strains were cultured in 43°C (non-permissive temperature for the replication of pMAD) to promote the first homologous recombination, followed by culturing 37°C to promote the second recombination, resulting in allelic exchange as described previously^22^.

### Genomic DNA sequencing and analysis

Genomic DNA was isolated with a DNeasy Kit (Qiagen), and dsDNA was quantified with a Qubit HS Assay Kit (Invitrogen). Indexed, paired-end libraries were made from 1 ng samples of the MNKN recipient and the transductant with a Nextera XT DNA Sample Preparation Kit (Illumina). Libraries were cleaned with 1.2× AMPure XP beads (Agencourt) and sequenced using a 300 cycle MiSeq Reagent Kit v2 on an Illumina MiSeq instrument (Illumina). Using CLC Genomics Workbench v6, reads were trimmed and filtered for base quality, and assembled *de novo*. Recombined regions between the MNKN recipient and RF122 donor (GenBank NC_007622) were identified through local alignments.

## Author Contributions

K.S.S designed the research. B.Y.M., J.Y.P. and S.Y.H. conducted the experiments. D.A.R. and J.C.T. provided critical sequencing information. Y.H.P., J.C.T, J.R.F., G.A.B., D.A.R. and K.S.S. analyzed data and wrote the manuscript.

## Supplementary Material

Supplementary Information Supplementary Information

## Figures and Tables

**Table 1:** Transduction frequencies of φSaBov_N, _φSaBov_EGC, _and φSaBov_LUK_

		Transfer frequency (CFU/pfu)*		
Recipient lineage	Recipient strain name	φSaBov_N_	φSaBov_EGC_	φSaBov_LUKE_
ST36-SCC*mec*II(USA200)	MN PE	2.50 ×10^−7^	5.00 ×10^−8^	5.00 ×10^−8^
	MN Park	None	None	None
	MN White	None	None	None
	MN PAM	None	None	None
ST8-SCC*mec*IV(USA300)	DAR1809	1.15 ×10^−6^	3.00 ×10^−7^	1.00 ×10^−8^
	DAR2017	8.00 ×10^−7^	1.25 ×10^−7^	1.00 ×10^−8^
	DAR1085	5.00 ×10^−7^	None	None
	DAR1964	4.50 ×10^−7^	None	None
ST1-SCC*mec*IV(USA400)	MW2	1.85 ×10^−6^	3.00 ×10^−7^	1.50 ×10^−8^
	MN KN	9.38 ×10^−5^	4.80 ×10^−6^	1.00 ×10^−7^
	MN Gary	2.00 ×10^−5^	4.80 ×10^−6^	None
	C99-193	2.15 ×10^−6^	1.00 ×10^−7^	1.25 ×10^−8^
	C99-529	2.05 ×10^−6^	2.50 ×10^−7^	1.00 ×10^−8^
Bovine-CC151	CTH96	4.36 ×10^−4^	1.22 ×10^−5^	7.00 ×10^−6^

Table S4To determine transduction frequency of φSaBov_N_, transduction frequency of phages induced from RF122 *SAB1737*::*tetM* was subtracted by that of φSaBov_EGC_.
