# Supplementary material for: Phage-mediated horizontal transfer of a Staphylococcus aureus virulence-associated genomic island
Source: Sci Rep. 2015 Apr 20;5:9784. doi: 10.1038/srep09784 (PMC4402969; doi:10.1038/srep09784)
Supplement: Supplementary Information [file srep09784-s1.pdf]

Phage-mediated horizontal transfer of a *Staphylococcus aureus* virulence-associated genomic island

Bo Youn Moon[1, 2], Joo Youn Park[1], Sun Yung Hwang[2], D. Ashley Robinson[3], Jonathan C. Thomas[3], J. Ross Fitzgerald[4], Yong Ho Park[2], Keun Seok Seo[1*]

[1]Department of Basic Sciences, College of Veterinary Medicine, Mississippi State University, Mississippi state, MS 39762, United Sates

[2]Department of Microbiology, College of Veterinary Medicine and BK21 Program for Veterinary Science, Seoul National University, Seoul 151-742, South Korea

[3]Department of Microbiology, University of Mississippi Medical Center, Jackson, MS 39216, United States

[4]The Roslin Institute and Edinburgh Infectious Diseases, Royal (Dick) School of Veterinary Studies, University of Edinburgh, Midlothian EH259RG, United Kingdom

* Corresponding author

Correspondence and requests for materials should be addressed to Keun Seok Seo (email: seo@cvm.msstate.edu)

Supplementary Table S1. Bacterial strains used in this study

S. aureus strains	Description	Reference
RF122	Bovine mastitis isolate, CC151	[1,2]
RF122 *sem*::*tetM*	Indicative strain for transfer of ϕ$SaBov_{EGC}$	This study
RF122 *SAB1737*::*tetM*	Indicative strain for transfer of ϕ$SaBov_{N}$ and ϕ$SaBov_{EGC}$	This study
RF122 *lukE*::*tetM*	Indicative strain for transfer of ϕ$SaBov_{LUKE}$	This study
RF122 *sem::tetM, int::cat*	RF122 *sem::tetM* ΔInt	This study
RF122 *sem*::*tetM, terL::cat*	RF122 *sem::tetM* ΔTerL	This study
MN PE	Human MRSA USA200, ST36-SCCmecII	[1,2]
MN Park	Human MRSA USA200, ST36-SCCmecII	[1,2]
MN White	Human MRSA USA200, ST36-SCCmecII	[1,2]
MN PAM	Human MRSA USA200, ST36-SCCmecII	[1,2]
DAR1809	Human MRSA USA300, ST8-SCCmecIV	[3,4]
DAR2017	Human MRSA USA300, ST8-SCCmecIV	[3,4]
DAR1085	Human MRSA USA300, ST8-SCCmecIV	[3,4]
DAR1964	Human MRSA USA300, ST8-SCCmecIV	[3,4]
MW2	Human MRSA USA400, ST1-SCCmecIV	[5,6]
MN KN	Human MRSA USA400, ST1-SCCmecIV	[7,8]
MN Gary	Human MRSA USA400, ST1-SCCmecIV	[5,6]
C99-193	Human MRSA USA400, ST1-SCCmecIV	[5,8]
C99-529	Human MRSA USA400, ST1-SCCmecIV	[5,8]
CTH96	Bovine mastitis isolate, CC151	[9]

Supplementary Table S2. List of primers used in this study

Name	Sequences (5' to 3')
Probe synthesis for southern blot	
semf	GTGAAAAACTATTATTGTCAGGAT
semr	TTGGGTTAATGGCAACCATAAAACA
SAB1737f	GAACCAGTACGGATCACGTGAA
SAB1737r	GTTGAATATCAGCATTACGATGATGTC
Intf	CATCACTGGTGGACGCTTTG
Intr	AATGCATCGAGCGCTTTTTC
pLukD	CACTCGTATCACTTGAACCTTTTTCA
pLukE	GGTGGCAATGGCTCATTTAATT
Selective marker	
tetMf	GCGCGTCGACGATCAAGAAACAAAGGCAACCCA
tetMr	GCGCGAATTCTAGGACACAATATCCACTTGTAG
Catf	GCTAGTCGACACGAAAGTCGAAGGGGGTTTTTA
Catr	GCTAGAATTCGGCCCGGTACCCAGCTTTT
Allelic replacement of *sem,* RF122 *sem::tetM*	
semupf	GCGCGGATCCTCTTAAGTTACCTACACC
semupr	GCGCGTCGACGAAAATCATATCGCAACC
semdnf	GCGCGAATTCTATCAAGTTCTTGTGCAG
semdnr	GCGCAGATCTGTCTATTATCTGAGTCGC
Allelic replacement of untranslation region of SAB1737, RF122 *SAB1737::tetM*	
1737upf	GCGCGGATCCTTATGCTTCACTCCATTTC
1737upr	GCGCGTCGACATGGGCAGTGTTGTAATTAT
1738dnf	GCGCGAATTCTGTTGTTGCATTAAATCACT
1738dnr	GCGCAGATCTTGATATTTAGAGGTGGCACA
Allelic replacement of *lukE*, RF122 *lukE::tetM*	
LukDupf	GCGCGGATCCGCAGCCATCTCCAAATTC
LukDupr	GCGCGTCGACCTAATCCTGGGGTATAACTG
LukEdnf	TGATGAATTCCTATTGCCCGTTAAACGG
LukEdnr	ATTGAGATCTCCTGTCGGTTTACTCATTG
Generating integrase knock out strain, RF122 *sem::tetM* ΔInt	

Intupf	GCGCGGATCCGCTCCTTTACGGAGCTTTAA
Intupr	GCGCGTCGACAATAAGGGTAGGCGAGCTAC
Intdnf	GCGCGAATTCGCATATCTTGGGAACGTTTC
Intdnr	GCGCAGATCTAACAGAGAACATGTTGCTAC
Generating terminase knock out strain, RF122 *sem::tetM* ΔTerL	
Terupf	GCGCGGATCCTGTCAACATGGCTTTTTCTG
Terupr	GCGCGTCGACTTGCTGAGGGTCTTGTGTTC
Terdnf	GCGCGAATTCCTTTCCGACCACGGGTTAA
Terdnr	GCGCAGATCTACGAAAGTTTGCCGGAAATA
Outward PCR and sequencing	
pInt	CGAGATTTAACGAGGGATAGG
p1702	TTGACACTAGCTTTCCGTTG
p1693	CGATGTTAATGGTAGTGATCATGC
p1759	TTTTAGCTAGCGCGTTAGTG
Linear phage DNA fragment characterization	
p1651	TCGGCACCACAGTTTCATTC
p1654	ACCAACAGCACCAGCAATACG
p1655	CCATTTCAGCTTGATCACTCATACC
p1663	AACATATTTGTGGTCAGGAGCTGAA
p1664	GTTAATGCTCTTGGCGTACCAAT
p1675	TGAAGAAAGAAAATGTACCAGGAAATG
p1676	TTCTAAGCAAGCACTTACATTTGTACCT
p1691	ATCATAAGAAGAGAAGAACGAGCTAGACT
p1694	ATGGGATCGCAATACCACC
pseg	AGCAAGACACTGGCTCACTAA
Identification of ϕSaBov integration	
tRNAr	GTAGCAACATGTTCTCTGTT
Intf	AAAGCGCTCGATGCATTG
lukEf	CACTCGTATCACTTGAACCTTTTTCA
seif	TCAAGGTGATATTGGTGTAGGTAACTTAA
seir	AAGTGGCCCCTCCATACATG
1758f	CGAAGAATACGAATCAAAATCAGCTAA
1692r	AGCCGTTTCAGCTTGATATAACATAT

1760r	GAGCAATGGGTGTGTCTAATGC
Quantitative Real time PCR	
qrt intf	CAGCGTGAAGAAGAAAAGTTTATGAG
qrt intr	TAAGCGTTGTACTTCGCCAAAG
semf	GTGAAAAACTATTATTGTCAGGAT
semr	TTGGGTTAATGGCAACCATAAAACA
Verification of chromosomal DNA contamination in phage DNA preparation	
LPScoreF	TAAAGGTGCGGGAACTTTCG
LPScoreR	AAGCGAGATCATCTGCCGAG

Supplementary Table S3. Increased transduction frequencies of $\phi SaBov_{EGC}$ and $\phi SaBov_{LUK}$ in the intermediate forms of transductants

Recipient	Transfer frequency of $\phi SaBov_{EGC}$ (CFU/pfu)*
MW2	2.5×10^{-7}
MW2 carrying $\phi SaBov_{N}$	4.6×10^{-5}
MNKN	8.5×10^{-6}
MNKN carrying $\phi SaBov_{N}$	1.4×10^{-4}
RN4220	9.0×10^{-6}
RN4220 carrying $\phi SaBov_{N}$	6.0×10^{-5}
**Recipient**	**Transfer frequency of $\phi SaBov_{LUKE}$ (CFU/pfu)***
MW2	1.50×10^{-8}
MW2 carrying $\phi SaBov_{N}$ and $\phi SaBov_{EGC}$	8.50×10^{-7}

*The transduction frequency of $\phi SaBov_{EGC}$ and $\phi SaBov_{LUKE}$ was measured using phages induced from RF122 *sem*::*tetM* and RF122 *lukE*::*tetM*, respectively.

Supplementary Table S4. *spa* and MLST typing of bovine isolates harboring a phage insertion in the νSaβ.

						Alleles at indicated locus						
ID#	Ridom *spa* type[a]	eGenomics *spa* type[a]	eGenomics *spa* repeats	Clonal complex	Sequence type[b]	*arcC*	*aroE*	*glpF*	*gmk*	*pta*	*tpi*	*yqiL*
OSU40	new1	new2	NEW-J1-G1-F1-M1-B1-B1-B1-B1-P1-B1	97	352	3	78	1	1	1	5	3
OSU8	new2	new3	T1-J1-G1-F1-B1-B1-B1-P1-B1	97	2187	3	317	1	1	1	5	3
OSU7	new2	new3	T1-J1-G1-F1-B1-B1-B1-P1-B1	97	2187	3	317	1	1	1	5	3
OSU17	t3992	1319	T1-J1-G1-F1-M1-B1-B1-P1-B1	97	2187	3	317	1	1	1	5	3
OSU19	t2112	445	T1-J1-G1-F1-M1-B1-B1-B1-B1-P1-B1	97	2187	3	317	1	1	1	5	3
OSU14	t13401	new1	U1-J1-G1-F1-G1-F1-M1-B1-B1-B1-B1-P1-B1	97	2187	3	317	1	1	1	5	3
OSU16	t267	105	U1-J1-G1-F1-M1-B1-B1-B1-P1-B1	97	2187	3	317	1	1	1	5	3
OSU45	t529	102	Z1-B1	151	351	6	72	50	43	52	67	59
OSU85	t529	102	Z1-B1	151	351	6	72	50	43	52	67	59

[a] Ridom and eGenomics *spa* types determined using resources at www.spaserver.ridom.de and www.egenomics.com, respectively.

[b] Multilocus sequence types determined using resources as saureus.mlst.net.

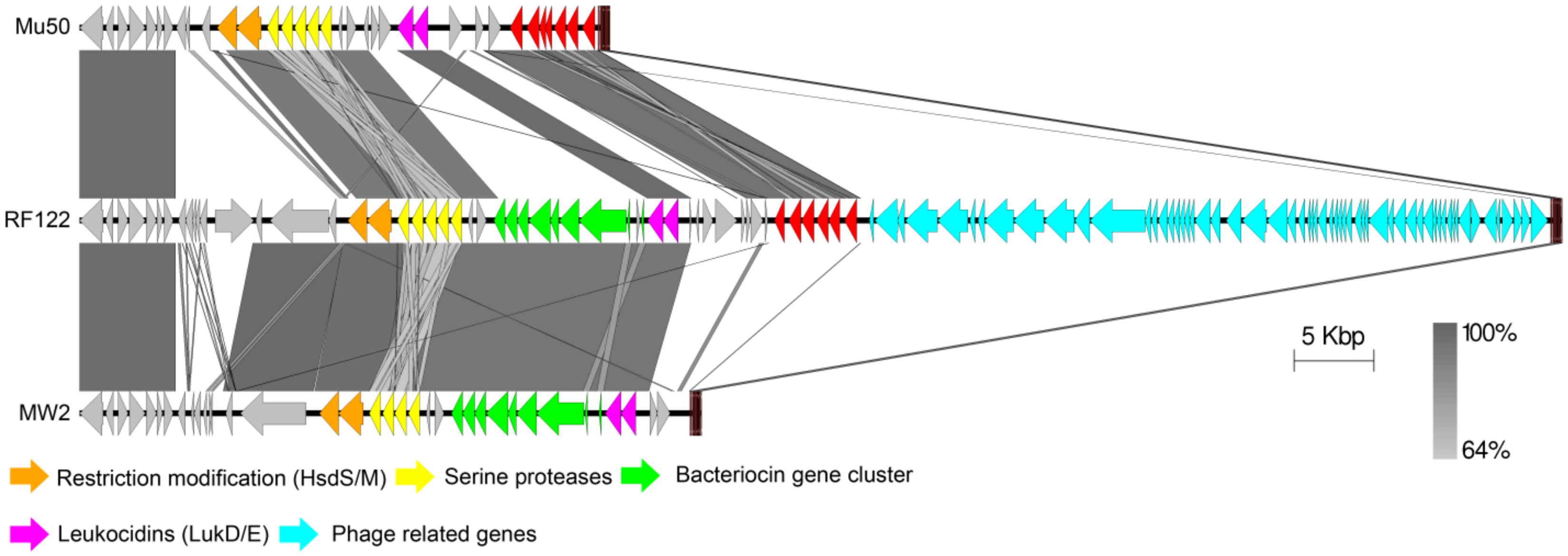

Supplementary Figure S1. Sequence alignment of νSaβ in Mu50, RF122, and MW2 strains. The arrow represents annotated genes in the entries [6,10,11] and colored based on key features. Orange; restriction modification system HsdR/M, yellow; serine protease cluster (*spl*), light green; bacteriocin gene cluster (*bsa*), pink; leukocidins (*lukD/E*), red; enterotoxin gene cluster (*egc*), cyan; genes related to phage. Note that the strain Mu50 harbors the egc but lacks a bacteriocin gene cluster; the strain MW3 harbors a bacteriocin gene clusters but lacks the egc. Uniquely, the strain RF122 harbors bacteriocin gene cluster and the egc, as well as the phage (ϕSaBov) insertion at the upstream of tRNA cluster (brown bar). The shading between the entries represents the percent identity (BLASTn) from 64 % (light gray) to 100 % (dark gray).

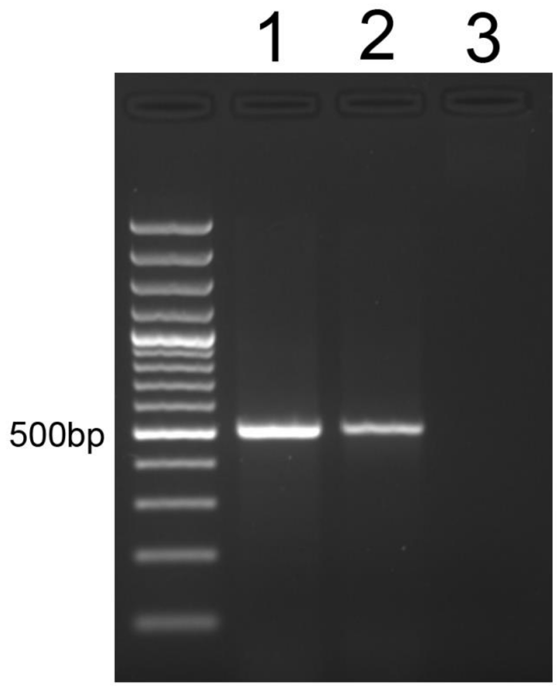

Supplementary Figure S2. Test for *S. aureus* chromosomal DNA contamination in the phage DNA preparation. Exogenous chromosomal DNA (*E. coli* DH5α, 10 μg) was added to the mitomycin C-induced culture lysate (10 ml) of the strain RF122, and then treated with excessive amount of RNase and DNAse I (100 unit each) for 2 hour, prior to the phage DNA extraction. PCR using primers specific to *E. coli* the *lps* gene (LPScoreF/R) was performed to check chromosomal DNA contamination in the phage DNA preparation. Results shown are PCR results with template using: Lane 1, purified *E. coli* chromosome; lane 2, *E. coli* chromosome added to mitomycin-induced culture lysate (before RNase and DNase I treatment); lane 3, phage DNA extraction after RNAse and DNAse treatments.

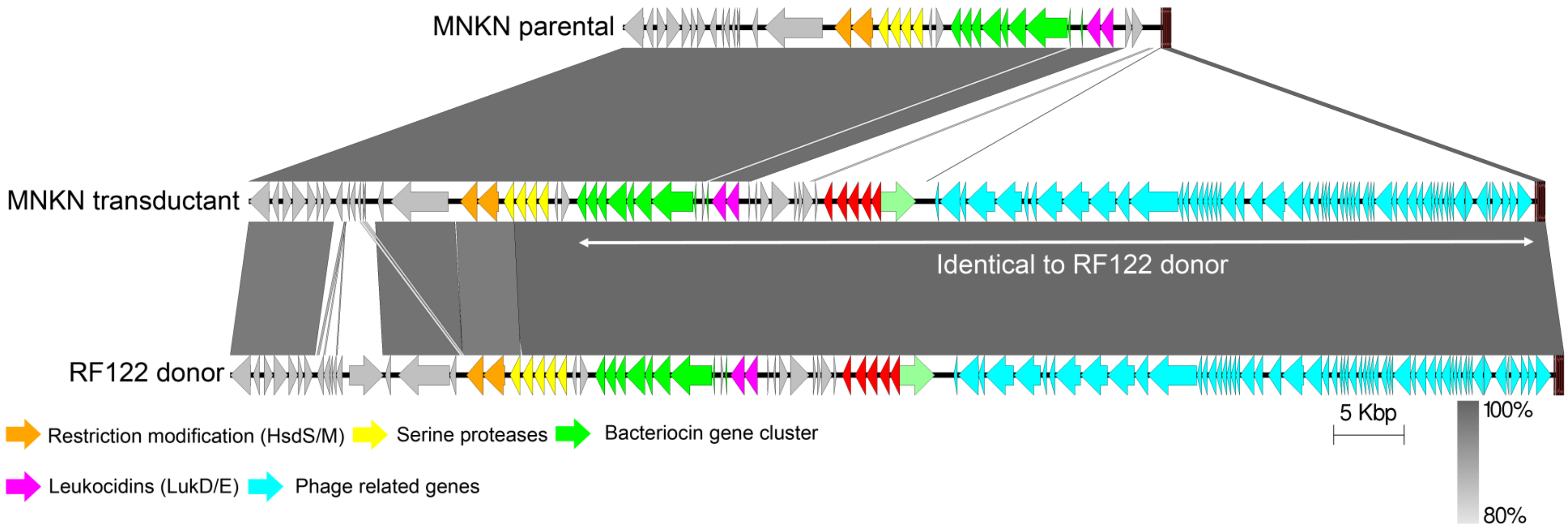

Supplementary Figure S3. Sequence alignment of νSAβ in MNKN recipient and transductant with RF122 donor. White arrow indicated the sequence identical between MNKN transductant and RF122 donor. The shading between the entries represents the percent identity (BLASTn) from 80 % (light gray) to 100 % (dark gray).

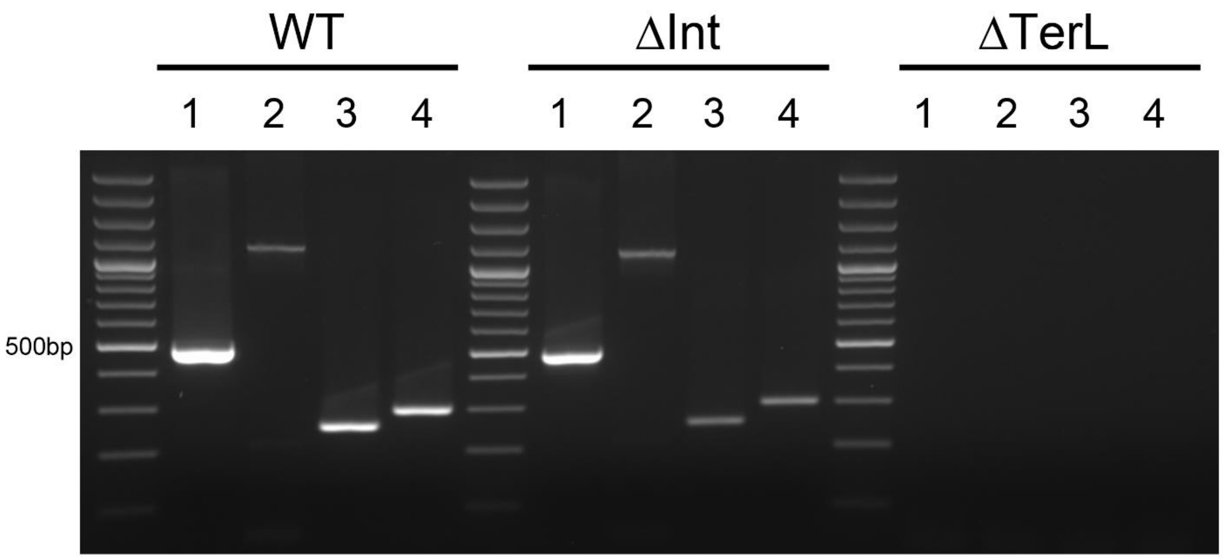

Supplementary Figure S4. The role of ϕSaBov encoded integrase and terminase on phage DNA excision and packaging. The integrase (SA1760) knock out (ΔInt) and terminase large subunit (SA1726) knock out (ΔTerL) strains were generated from RF122 *sem::tetM* by allelic replacement. Phage DNAs were extracted from these strains and tested by PCR and outward PCR as described in the text. Note that the ΔInt strain showed identical PCR product patterns to the wild type strain, indicating a disruption of the integrase gene did not affect phage DNA excision and packaging. However, the ΔTerL strain did not show any PCR products, most likely due to the inability to package phage DNA. Lane 1, outward PCR using primers pInt/p1702 (a marker for circular phage DNA of $\phi SaBov_{N}$); lane 2, outward PCR using primers p1693/p1759 (a marker for circular phage DNA of $\phi SaBov_{EGC}$); lane 3, PCR using primers semf/semr (a marker for $\phi SaBov_{EGC}$); lane 4, PCR using primers pLukD/pLukE (a marker for $\phi SaBov_{LUKE}$)

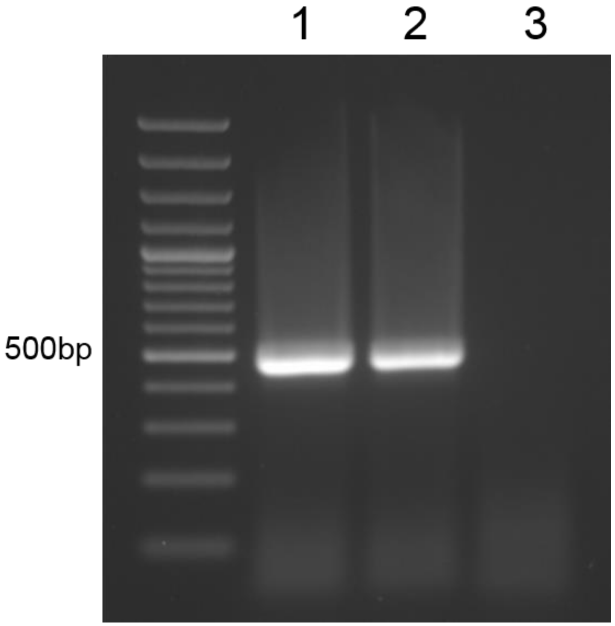

Supplementary Figure S5. The excision and circularization of ϕSaBov$_N$ is dependent on host background. RF122 and RN4220 and MW2 strains carrying ϕSaBov$_N$ were treated with mitomycin C and phage DNAs were extracted. Results shown are outward PCR with pIntF/p1702R primer set (a marker for circular phage DNA of ϕSaBov$_N$) using phage DNA template extracted from: Lane 1, RF122; lane 2, RN4220 carrying ϕSaBov$_N$; lane 3, MW2 carrying ϕSaBov$_N$. Note that the excision and circularization of the ϕSaBov$_N$ phage DNA was observed in RF122 and RN4220 carrying the ϕSaBov$_N$, not in MW2 strain carrying the ϕSaBov$_N$, indicating the excision and circularization of the ϕSaBov$_N$ phage DNA is dependent on host background.

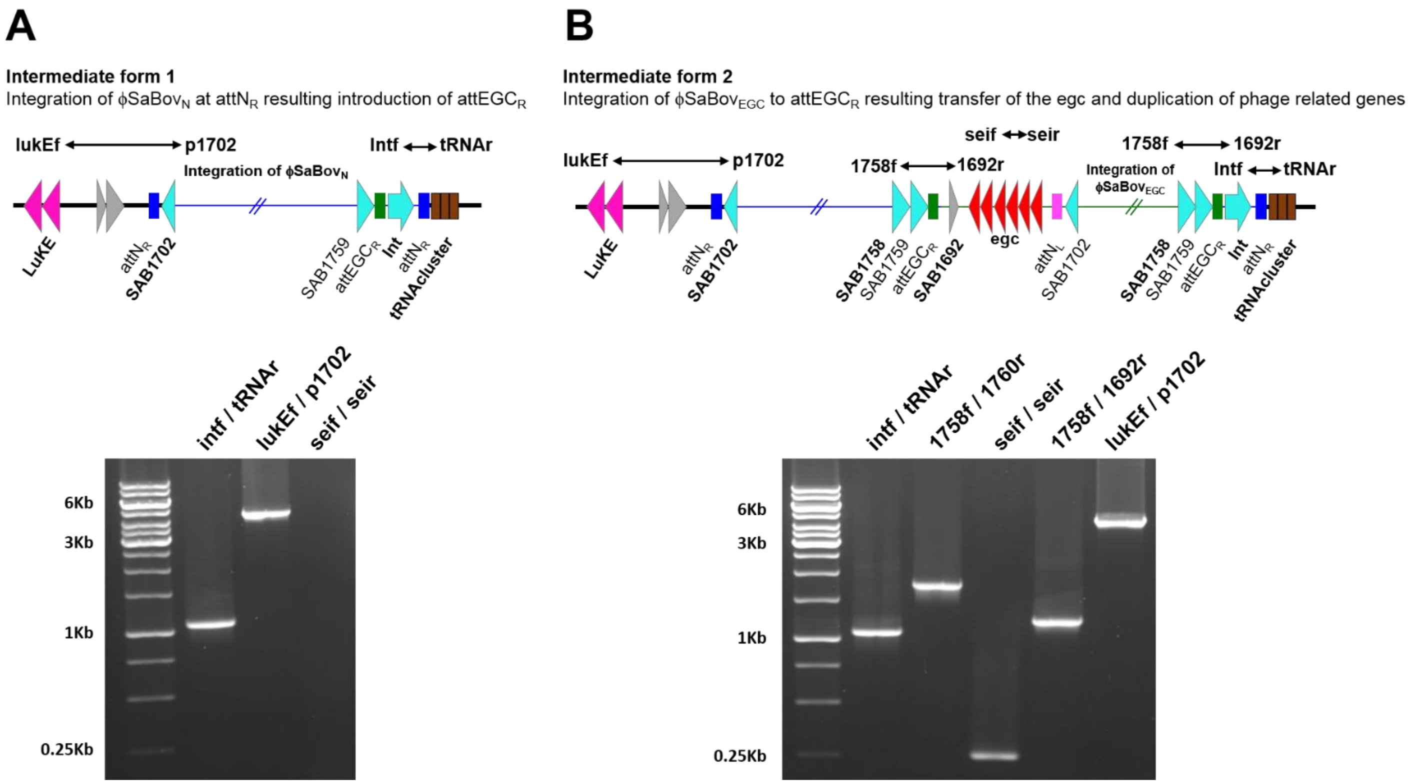

Supplemental Figure S6. Identification of intermediate forms of transductants. The MW2 strain transduced with phage induced from RF122 was randomly selected and screened by junction PCR. (A) Top panel: A schematic map of intermediate form of transductant just carrying the $\phi SaBov_N$ at the tRNA cluster and locations of primer set for junction PCR. Bottom panel: Results of junction PCR were shown. PCR amplicons with intf/tRNAr and lukEf/p1702 primer sets and no amplicon with seif/seir primer set indicate $\phi SaBov_N$ was integrated at the $attN_R$ but not $\phi SaBov_{EGC}$. (B) Top panel: A schematic map of intermediate form of transductant harboring the integration of $\phi SaBov_{EGC}$ at the $attEGC_R$, resulting transfer of the egc and duplication of phage related genes. Bottom panel: Results of junction PCR were shown. PCR amplicons with 1758f/1692r, seif/seir, and 1758/1692r primer sets indicate the integration of the $\phi SaBov_{EGC}$ at the $attEGC_R$ and transfer of the egc. PCR amplicon with lukEf/p1702 primer set indicates the duplication of phage related genes.

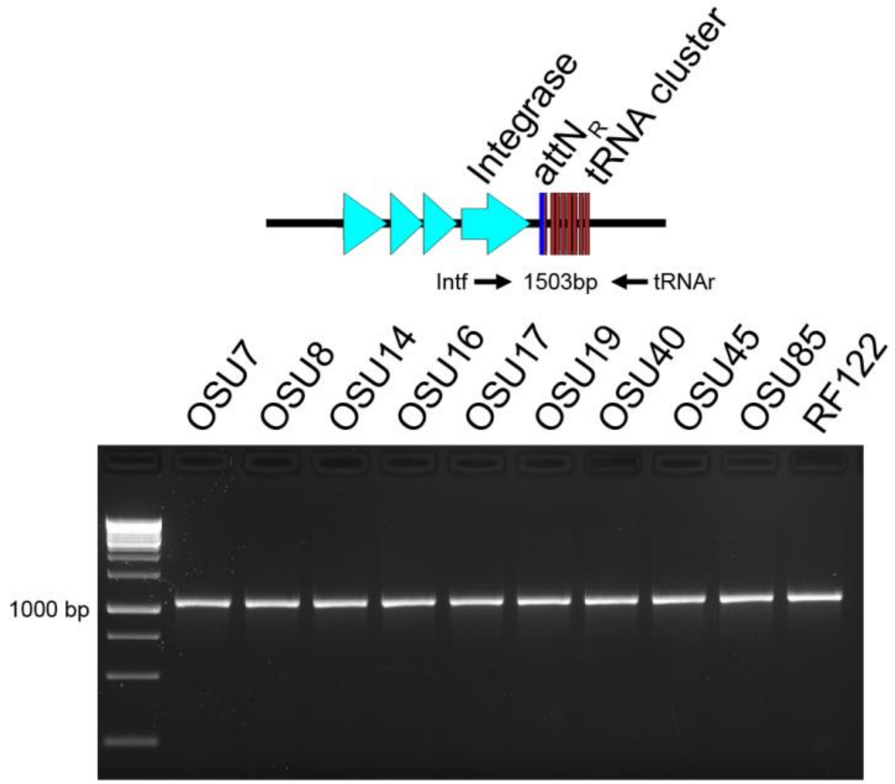

Supplementary Figure S7. Screening of ϕSaBov in bovine mastitis isolates. The insertion of phage in the νSaβ was screened by a primer pair as depicted in the figure. From a collection of 53 bovine mastitis strains isolated from Ohio state, USA from 2010 to 2013, 9 strains have a phage insertion at the same location in the strain RF122 as indicated by PCR results.

References

1 MacDonald, K. L. *et al.* Toxic shock syndrome. A newly recognized complication of influenza and influenzalike illness. *JAMA* **257**, 1053-1058 (1987).

2 Schlievert, P. M., Strandberg, K. L., Lin, Y. C., Peterson, M. L. & Leung, D. Y. Secreted virulence factor comparison between methicillin-resistant and methicillin-sensitive *Staphylococcus aureus*, and its relevance to atopic dermatitis. *J Allergy Clin Immunol* **125**, 39-49 (2010).

3 Robinson, D. A. *et al.* Re-emergence of early pandemic *Staphylococcus aureus* as a community-acquired meticillin-resistant clone. *Lancet* **365**, 1256-1258 (2005).

4 Robinson, D. A. & Enright, M. C. Multilocus sequence typing and the evolution of methicillin-resistant *Staphylococcus aureus*. *Clin Microbiol Infect* **10**, 92-97 (2004).

5 Prevention, C. f. D. C. a. Four pediatric deaths from community-acquired methicillin-resistant *Staphylococcus aureus* - Minnesota and North Dakota, 1997-1999. Report No. 0149-2195 (Print)

0149-2195 (Linking), 707-710 (1999).

6 Baba, T. *et al.* Genome and virulence determinants of high virulence community-acquired MRSA. *Lancet* **359**, 1819-1827 (2002).

7 Rizkallah, M. F., Tolaymat, A., Martinez, J. S., Schlievert, P. M. & Ayoub, E. M. Toxic shock syndrome caused by a strain of *Staphylococcus aureus* that produces enterotoxin C but not toxic shock syndrome toxin-1. *Am J Dis Child* **143**, 848-849 (1989).

8 Fey, P. D. *et al.* Comparative molecular analysis of community- or hospital-acquired methicillin-resistant *Staphylococcus aureus*. *Antimicrob Agents Chemother* **47**, 196-203 (2003).

9 Joo, Y. S., Fox, L. K., Davis, W. C., Bohach, G. A. & Park, Y. H. *Staphylococcus aureus* associated with mammary glands of cows: genotyping to distinguish different strains among herds. *Vet Microbiol* **80**, 131-138 (2001).

10 Herron-Olson, L., Fitzgerald, J. R., Musser, J. M. & Kapur, V. Molecular correlates of host specialization in *Staphylococcus aureus*. *PLoS One* **2**, e1120 (2007).

11 Ohta, T. *et al.* Nucleotide substitutions in *Staphylococcus aureus* strains, Mu50, Mu3, and N315. *DNA Res* **11**, 51-56 (2004).
